# Antimicrobial stewardship situation analysis in selected hospitals in Zambia: findings and implications from a national survey

**DOI:** 10.3389/fpubh.2024.1367703

**Published:** 2024-09-27

**Authors:** Joseph Yamweka Chizimu, Steward Mudenda, Kaunda Yamba, Chileshe Lukwesa, Raphael Chanda, Ruth Nakazwe, Bwalya Simunyola, Misheck Shawa, Aubrey Chichonyi Kalungia, Duncan Chanda, Uchizi Chola, Tebuho Mateele, Jeewan Thapa, Kenneth Kapolowe, Mazyanga Lucy Mazaba, Mirfin Mpundu, Freddie Masaninga, Khalid Azam, Chie Nakajima, Yasuhiko Suzuki, Nathan Nsubuga Bakyaita, Evelyn Wesangula, Martin Matu, Roma Chilengi

**Affiliations:** ^1^Antimicrobial Resistance Coordinating Committee (AMRCC), Zambia National Public Health Institute, Lusaka, Zambia; ^2^Division of Bioresources, Hokkaido University International Institute for Zoonosis Control, Sapporo, Japan; ^3^Department of Pharmacy, School of Health Sciences, University of Zambia, Lusaka, Zambia; ^4^Lusaka District Health Office, Lusaka, Zambia; ^5^University Teaching Hospitals, Lusaka, Zambia; ^6^Hokudai Center for Zoonosis Control in Zambia, Hokkaido University, Lusaka, Zambia; ^7^Department of Pharmacy, Ministry of Health, Lusaka, Zambia; ^8^Levy Mwanawasa University Teaching Hospital, Lusaka, Zambia; ^9^Division of Research Support, Hokkaido University Institute for Vaccine Research and Development, Kita-ku, Sapporo, Hokkaido, Japan; ^10^Action on Antibiotic Resistance (ReAct) Africa, Lusaka, Zambia; ^11^World Health Organization, Lusaka, Zambia; ^12^Strengthening Pandemic Preparedness, Eastern, Central, and Southern Africa Health Community, Arusha, Tanzania; ^13^International Collaboration Unit, Hokkaido University International Institute for Zoonosis Control, Sapporo, Japan

**Keywords:** antimicrobial resistance, antimicrobial stewardship, core elements, situation analysis, Zambia

## Abstract

**Background:**

Antimicrobial stewardship (AMS) programs are critical in combating antimicrobial resistance (AMR). In Zambia, there is little information regarding the capacity of hospitals to establish and implement AMS programs. The objective of this study was to conduct a baseline assessment of WHO core elements for an AMS program implementation in eight hospitals in Zambia.

**Materials and methods:**

We conducted an exploratory cross-sectional study from September 2023 to December 2023 using a self-scoring Periodic National and Healthcare Facility Assessment Tool from the World Health Organization (WHO) policy guidance on integrated AMS activities in human health. Eight public hospitals were surveyed across the five provinces of Zambia. Data was analyzed using the WHO self-scoring tool and thematic analysis.

**Results:**

Overall, 62.5% (6/8) of the facilities scored low (below 60%) in implementing AMS programs. Most facilities had challenges with reporting AMS feedback within the hospital (average score = 46%), Drugs and Therapeutics Committee (DTC) functionality (average score = 49%), AMS actions (average score = 50%), education and training (average score = 54%), and leadership commitment to AMS activities (average score = 56%). The overall score for all AMS core elements was average (56%). All the hospitals (100%) did not have an allocated budget for AMS programs. Finally, there were neither antibiograms to guide antimicrobial utilization nor AMS-trained staff in more than 50% of the hospitals surveyed.

**Conclusion:**

This study found low AMS implementation in these public hospitals, especially where DTCs were non-functional. The identified challenges and gaps require urgent attention for sustainable multidisciplinary AMS programs.

## Introduction

Antimicrobial resistance (AMR) is the world’s most pressing public health problem affecting all countries (1–3). This problem has been exacerbated by the overuse and misuse of antimicrobials in humans, animals, and the environment (3, 4). Unfortunately, drug-resistant pathogens cause infections that may be difficult or impossible to treat (5–7). This may lead to prolonged hospital stays, increased medical costs, and increased mortality (2, 8, 9). Alongside this, AMR negatively impacts the global economy (10, 11). If this problem is unchecked, it will lead to more than 10 million human deaths annually by the year 2050 and extreme poverty for an additional more than 20 million people (4, 12, 13). Hence, key stakeholders across the globe are playing critical roles in the mitigation of AMR (14, 15). Importantly, these stakeholders include healthcare workers (HCWs) who must ensure the prudent use of antibiotics to prevent the emergence and spread of antibiotic-resistant pathogens (16, 17).

Therefore, addressing AMR requires strategies that promote the prudent use of available antibiotics (18–21). One important strategy for addressing AMR is the establishment and implementation of antimicrobial stewardship (AMS) programs (20, 22, 23). These are coordinated programs that promote the appropriate use of antimicrobials thereby improving patient outcomes, reducing microbial resistance, reducing costs, and decreasing the spread of infections caused by multidrug-resistant organisms (22, 24–28). AMS programs provide guidelines on the rational use of antibiotics and ensure that antibiotics are prescribed to the right patient, in the right dose, at the right time, in the right frequency, and for the right duration (29–36). Notably, it is documented that instigating AMS in healthcare facilities facilitates the prevention of healthcare-acquired and multidrug-resistant infections (37–42).

Additionally, AMS programs include educational and sensitization activities for HCWs on rational prescribing, dispensing, and use of antibiotics (29–35). In Ghana, a study on AMS reported that HCWs were highly aware of AMR influencing them to prescribe, dispense, and administer antibiotics rationally (43). Interestingly, AMS programs also involve patient and community education (18, 27, 44–46). This promotes the rational use of antibiotics among patients and discourages self-medication practices in communities (47–50). Moreover, evidence has shown that knowledge and awareness of AMR are also a contributing factor to the rise of this problem (51–56) as individuals who are not aware of AMR tend to overuse and misuse antibiotics (52, 57, 58).

In 2017, the World Health Organization (WHO) developed the Access, Watch, and Reserve (AWaRe) classification of antibiotics as a tool for AMS to monitor the prescribing and use of antibiotics in healthcare facilities (59, 60). The WHO AWaRe framework stipulates that most antibiotics (60%) prescribed in healthcare facilities must belong to the Access group to reduce the emergence of resistance to other categories of antibiotics (37, 61, 62). The Access group includes antibiotics that exhibit lesser potential for resistance while demonstrating effectiveness against a broad spectrum of frequently encountered susceptible infections (63, 64). Alongside this, the Access group comprises narrow-spectrum antibiotics used as the first and second choice for common infections including respiratory tract and ear infections (37, 65). Subsequently, the WHO recommended adherence to the AWaRe framework to address AMR (64, 66–69). The Watch group category contains generally broad-spectrum antibiotics that have been recommended only for specific indications because of their high potential to develop resistance (63, 64, 70). The Reserve group category includes antibiotics that are used as a last resort for the treatment of multidrug-resistant pathogens (61, 71). Non-adherence to the AWaRe framework may also contribute to the inappropriate prescribing, dispensing, and administration of antibiotics (72). Moreover, the WHO has also provided the core elements as benchmarks against which functional AMS programs can be evaluated for their ability to successfully support the appropriate use of antibiotics in hospitals (73–75).

Based on the WHO core elements, most high-income and developed countries have successfully implemented AMS programs which have led to a reduction in the incidence of AMR (76). Nevertheless, there is currently insufficient data demonstrating the extent and implementation of AMS programs, especially in low-income countries like Zambia, with documented challenges (77). Studies conducted in some healthcare facilities in Zambia have reported that most prescribed and dispensed antibiotics belong to the Watch group with high empiric prescribing and limited documentation of the rationale behind antibiotic prescribing (68, 69, 78). This evidence shows deviations from the WHO recommendations on rational prescribing and use of antibiotics in hospitals (61, 63–65).

Hence, Zambia developed its Multisectoral National Action Plan (NAP) on AMR in 2017 to provide a coherent framework for combating AMR through the One Health approach (79). One of the five strategic objectives of the NAP is to optimize the use of antimicrobials in human, animal, and plant health through AMS programs (79). Besides, a few cooperating partners started implementing some AMR activities in selected facilities. Nevertheless, there is still a paucity of information concerning adherence to WHO facility AMS core elements. The core elements include healthcare facility leadership commitment, presence of Drugs or Medicines and Therapeutics Committees (DTC), AMS and IPC teams, accountability and responsibility, AMS actions, education and training, monitoring and surveillance, and reporting feedback on AMS activities within healthcare facilities. It is with this background that we conducted a situation analysis that assessed the state of AMS implementation in selected public hospitals in Zambia using the WHO guidelines on integrated AMS activities in human health. This was premised to be a starting point towards quality improvement and building a case for AMS mainstreaming for stakeholder action.

## Materials and methods

### Study design, site, and population

**Figure 1:**
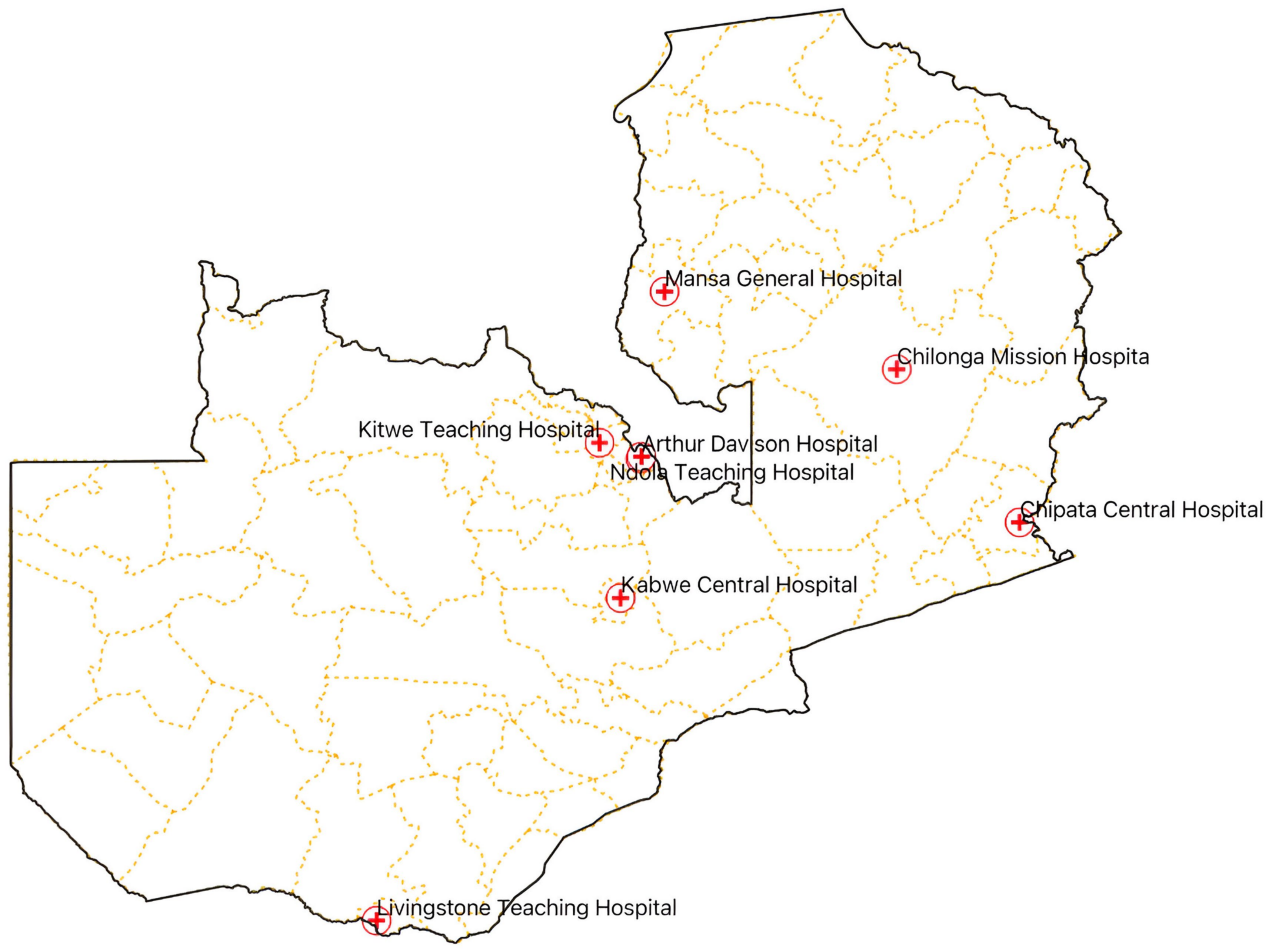
Map of Zambia indicating the surveyed healthcare facilities.

We conducted an exploratory cross-sectional study from September to December 2023, across eight hospitals in Zambia, namely, the Arthur Davison Children’s Hospital (ADH), Ndola Teaching Hospital (NTH), Kitwe Teaching Hospital (KTH) in the Copperbelt Province, Chilonga Mission Hospital (CMH) in Muchinga Province, Chipata Central Hospital (CCH) in the Eastern Province, Kabwe Central Hospital (KCH) in the Central Province, Livingstone Central Hospital (LCH) in the Southern Province, and Mansa General Hospital (MGH) in the Luapula Province (Figure 1). These healthcare facilities were chosen as they are the referral hospitals in the selected six out of the ten provinces in Zambia with high hospital annual admissions ranging from 3,500 to 19,656 patients. The number of bed spaces for these hospitals ranged from a minimum of 230 to a maximum of 741 as shown in Table 1. The facilities were also considered because of their ability to provide minimum to advanced laboratory testing to support AMS programs compared to other hospitals in the country. The selected hospitals were the sentinel sites for AMR surveillance in the country though they did not have established AMS programs. Therefore, the baseline assessment was conducted in these AMR surveillance sites to investigate their capacity to implement AMS programs. The participants of this study included domiciled healthcare workers who were members of the respective hospitals’ AMS, DTC, or IPC teams inclusive of pharmacists (25%), physicians (25%), environmental health technologists (9%), laboratory scientists (22%), and nurses (19%) as shown in Table 2. Although self-treatment and sale of antibiotics without prescriptions in informal and retail pharmacies are major contributors to the development of AMR, this study only targeted hospitals and excluded retail or informal pharmacies.

**Table 1:** Demographic profiles for the public hospitals included in this study.

Level of care	Public hospital name	Province	No: bed spaces	Hospital annual admissions
Tertiary	Ndola Teaching	Copperbelt	741	19,656
	Kitwe Teaching	Copperbelt	500	15,101
	Arthur Davidson Children’s Teaching	Copperbelt	250	10,800
	Livingstone Teaching	Southern	325	10,871
	Chipata Central	Eastern	600	11,620
	Kabwe Central	Central	474	14,940
Secondary	Mansa General	Luapula	420	4,908
	Chilonga Mission General	Muchinga	230	3,500

**Table 2:** Sociodemographic characteristics of the multi-disciplinary key informants.

		Frequency	Percent
Gender	Female	20	63
	Male	12	38
	Pharmacists	8	25
Profession	Environmental technologist	3	9
	Clinicians	8	25
	Nurses	6	19
	Laboratory personnel	7	22

### Sample size estimation and sampling criteria

To get a representative sample, we interviewed four members of the AMS team, DTC, or ICC per healthcare facility. This translated into a minimum sample of 32 respondents. All participants were selected using a purposive sampling method because they were the best source of information regarding AMR activities in the selected healthcare facilities. Purposive sampling is useful when enrolling participants or institutions with specific characteristics (80). Therefore, we used a purposive sampling method to identify and enrol specific healthcare workers who are members of the DTC, ICC, or AMS committees. Additionally, these participants were from multi-disciplinary professions involving Pharmacists, Nurses, Clinicians, Laboratory personnel, and Environmental technologists.

### Data collection

Data were collected using the validated Periodic National and Healthcare Facility Assessment Tool in the WHO policy guidance on integrated AMS activities in human health (81). Using this tool, healthcare facilities can assess their level of preparedness for AMS about their core elements, create a step-by-step implementation plan, and track their progress over time in implementing AMS programs and activities (81). The tool was developed by the WHO to guide member states on how to implement AMS activities in a programmatic and integrated approach. It complements the Global Action Plan on AMR and the WHO toolkit to address AMR in low-and middle-income countries (82, 83). The assessment tool assesses healthcare facility AMS using six core elements with each having components of assessments (indicators). The core elements included; General [presence of DTC, Infection Control Committee (ICC), and AMS teams]; DTC functionality (9 indicators); Leadership commitment (6 indicators); accountability and responsibility (10 indicators); AMS Actions (11 indicators); Education and Training (3 indicators); Monitoring and surveillance (6 indicators); and Reporting Feedback within the Healthcare Facility (four indicators) (81).

The responses were scored as 0 = No, 1 = No but a priority, 2 = Planned but not started, 3 = Partially implemented, 4 = Yes (Fully implemented). The overall score of 80–100% indicated that AMS was fully implemented and functioning well, but needed continuous support for sustainability, 50–79.9%, AMS was partially functioning and needed attention for strengthening while 0.0–49.9% indicated that AMS was poorly functioning or non-functioning, and needed prioritized attention (81, 84). The tool was reviewed by members of the National AMS Technical Working Group (TWG) under Antimicrobial Resistance Coordinating Committee (AMRCC) at the Zambia National Public Health Institute (ZNPHI). No modifications were made to the tool.

The data collectors were also members of AMS National TWG. These were trained on how to administer the interview, sticking to the questions in the questionnaire, the importance of neutrality, and reducing bias. Additionally, role-playing exercises were carried out to help interviewers refine their ability to ask consistent and unbiased questions. Further, the three interviewers were staff from diverse backgrounds including nurses, clinicians, pharmacists, and laboratory staff. To familiarize the data collectors with the data collection tool, we conducted pilot studies in two hospitals in the Lusaka District which were excluded from the assessment. The face-to-face interviews were conducted by three data collectors per healthcare facility. All the data were collected using tablets and computers with restricted access to authorized personnel. Data collection took place for 2 days per hospital and proceeded without challenges.

### Data analysis

Data were entered in the WHO self-scoring assessment tool in Microsoft Excel. Descriptive analysis was used to analyse the data using the WHO-validated self-scoring tool. The self-scoring tool provided a summary score of the six core elements of the assessment. This was followed by categorizing all responses into six themes under each core element including (i) Presence of DTC, ICC, AMS teams in surveyed hospitals, and DTC functionality, (ii) Leadership commitment, accountability, and responsibility, (iii) Antimicrobial stewardship actions, (iv) Education and training on AMS, (v) Monitoring and Surveillance, and (vi) Reporting feedback within the healthcare facility. Each of the core elements of AMS had an overall score that was calculated out of 100%. Interpretation of the findings was performed based on the Periodic National and Healthcare Facility Assessment Tool in the WHO policy guidance on integrated AMS activities in human health (81).

### Ethical considerations

Ethical approval was obtained from the Tropical Diseases Research Centre (TDRC) Ethics Committee with an approval number of TRC/C4/09/2023. Permission to access the respective hospital was obtained from the Hospital Management Teams. Furthermore, informed consent was acquired from the hospital leadership and key informants. To preserve anonymity and protect hospital staff who took part in the study from any unfavorable outcomes, no personal identity information was collected.

## Results

### Baseline information on AMS in surveyed hospitals

**Figure 2:**
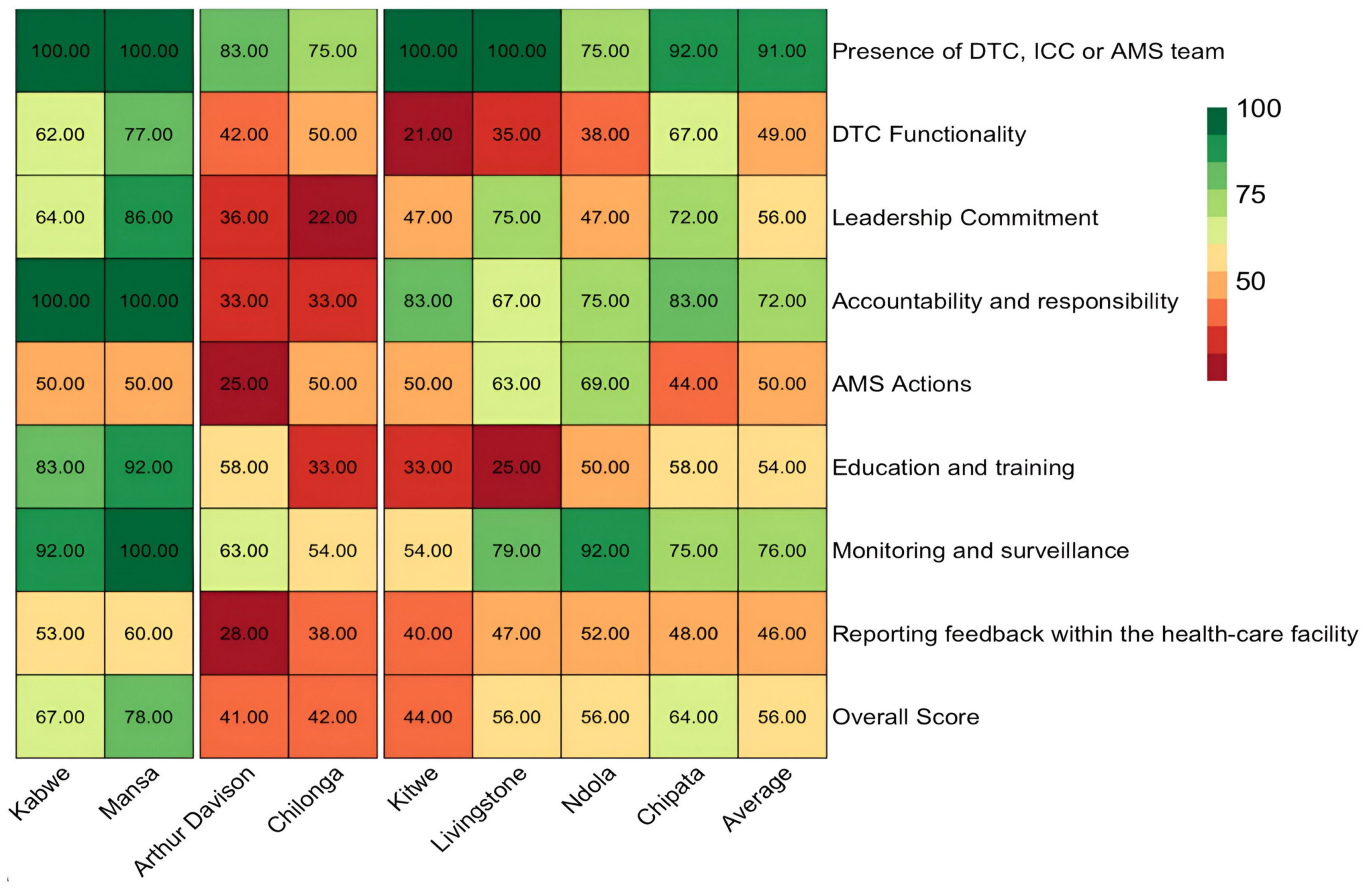
Heatmap showing the summary of scores regarding the core elements of AMS in surveyed hospitals. ICC, infection control committee.

This study found an average score of 56% for all AMS core elements in the priority healthcare facilities surveyed. MGH, KGH, LTH, NTH, and CCH had AMS programs partially functioning with scores of 56–78% and hence needed attention for strengthening while ADH, CMH, and KTH were poorly functioning, with scores of 41–44% and therefore, needed prioritized attention. The lowest scores on AMS activities were observed with ADH (41%), CMH (42%), KTH (44%), LTH (56%), and NTH (56%). The highest score in AMS core elements was recorded at MCH (78%) followed by KCH (67%). Therefore, no facility had an overall score above 80% to indicate a functioning AMS program. Overall, the assessment revealed the low performance of healthcare facilities on core elements such as DTC functionality (49%), leadership and commitment (56%), AMS actions (50%), education and training (54%), and reporting feedback within the healthcare facilities (46%) (Figure 2).

### Presence of DTC, ICC, AMS, and DTC functionality in surveyed hospitals

This indicator assessed the existence of the DTCs, AMS, or ICC and their functionality. Some of the indicators used to establish the functionality of these committees were the availability of regular meeting minutes and an antibiotic policy and procedure document, the presence of developed action plans that have been approved by management, and the categorization of antibiotics by the AWaRe classification.

Hence, the average score for the presence of DTC, AMS, and ICC was 91%, of which most facilities scored above 80%, with only two facilities scoring 75%. Nevertheless, the functionality of these committees was suboptimal with an average score of 49%. CCH and NTH did not have a fully functional IPC while ADH had no IPC committee (Supplementary Table S1). Similarly, ADH and Chilonga did not have AMS committees but had the DTC/MTCs (Supplementary Table S1).

Of the assessed facilities, none of the hospital DTCs had approved terms of reference, members assigned officially nor conducted supply and medicine use problem studies. Only KGH and MGH took action based on the findings from the supply and medicine study (scored 4).

**Figure 3:**
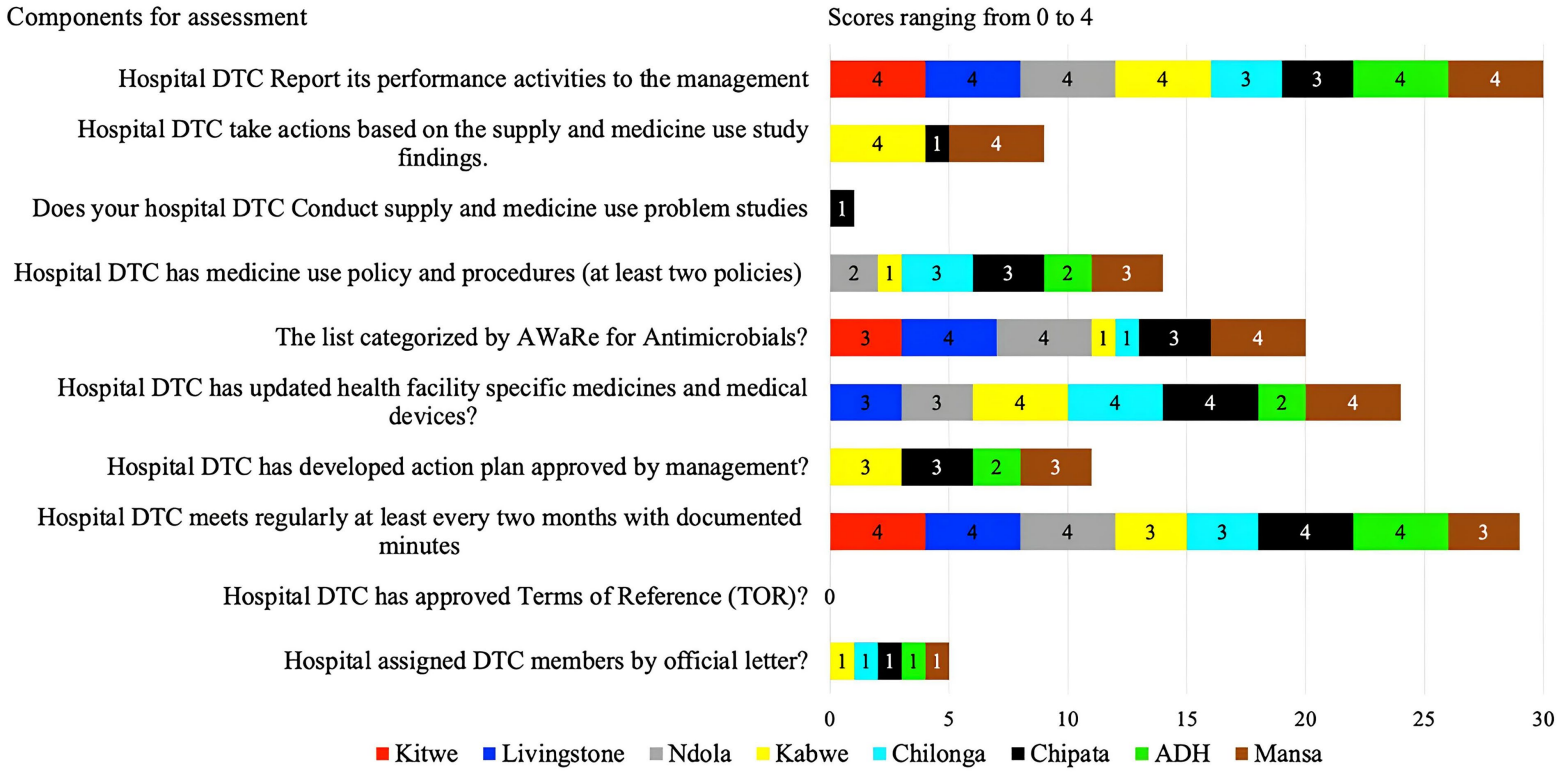
Scores of the eight facilities on their performance regarding DTC functionality.

KGH, CCH, and MGH had partially developed (score of 3) and none had fully developed DTC or AMS action plans (Figure 3). Whereas, ADH had planned for it (score of 2) but had not developed it. Three facilities (LTH, NTH, and MGH) had their specific health facility medicine lists categorized by AWaRe (score of 4) while the other three (ADH, CMH, and KGH) did not. Antimicrobial use policies and procedures were absent in all eight facilities with only CMH, MGH and CCH having partially developed (score of 3). However, facilities (KTH, LTH, NTH, KGH, ADH, and MGH) fully implemented hospital DTCs reporting their activities to management. Additionally, KTH, LTH, NTH, CCH, and ADH conducted regular meetings with documented minutes while CMH, CCH, and MGH partially implemented this. Further, four facilities (KGH, CMH, CCH, and MGH) had updated facility-specific medicines and medical devices while LTH and NTH partially implemented these indicators (Figure 3).

### Leadership commitment, accountability, and responsibility in surveyed hospitals

Leadership commitment to AMS involved prioritizing AMS programs by the leadership of the surveyed hospitals. This included supporting the DTC/AMS teams by allocating necessary human, financial, and information technology resources to AMS activities, as outlined in an endorsed institutional action plan. Whereas, accountability and responsibility involved the hospital having a single leader responsible for program outcomes on AMS. The leader or champion must have dedicated staff time for AMS activities in their terms of reference (TORs) or job description.

In this study, 50% (4/8) of facilities, namely ADH, KTH, CMH, and NTH, scored below average (56%) on leadership commitment to AMS activities (Figure 2). Nevertheless, AMS was considered a priority (score of 4) by the majority (6/8, 75%) of the healthcare facility management including NTH, KTH, LTH, CCH, MGH, and KGH. Four facilities (NTH, KTH, ADH, and CMH) had no AMS activities in their institutional annual action plans with key performance indicators. Additionally, there was no dedicated financial support for the healthcare facility AMS action plan in any of the assessed hospitals (Supplementary Table S1).

The presence of multidisciplinary AMS committee leadership in the healthcare facility with clear TORs was absent in four facilities (LTH, NTH, CMH, and ADH), present in two (MGH, KCH), and partially implemented in two facilities (KTH and CCH). Two facilities (MGH, and LTH) had a dedicated AMS leader/champion though none had dedicated staff time for AMS activities in their TORs or job descriptions. Additionally, two facilities (ADH, and CMH) reported not disseminating the AMS activity report to facility management and other healthcare facility team members (Supplementary Table S1).

### Antimicrobial stewardship actions in surveyed hospitals

AMS actions included target issues or activities undertaken by the hospital AMS program.

These were assessed based on the availability of updated Standard Treatment Guidelines (STGs), regular auditing of antibiotic therapy, conducting regular ward rounds on AMS, AWaRe classification of antibiotics in the hospital formulary, access to an information management system for data collection by the AMS team, and a written AMS policy requiring prescribers to indicate the reason for antibiotic prescriptions.

**Figure 4:**
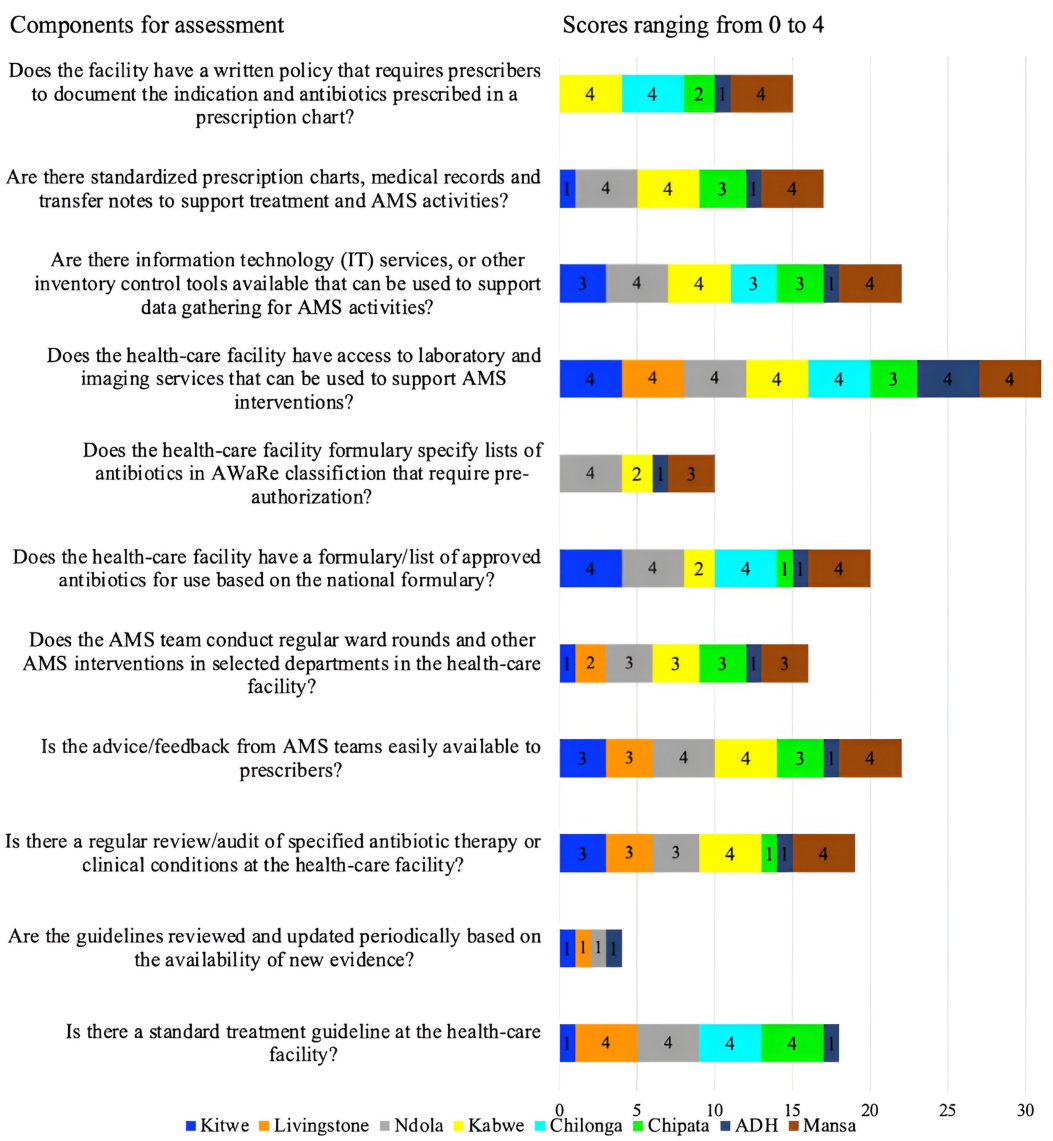
Scores of the eight facilities on their performance regarding AMS actions.

Overall, 25% (2/8) of the hospitals (ADH and CCH) scored below average (50%), while 50% (4/8, KCH, MCH, CMH, and KTH) scored 50% due to a lack of comprehensive AMS actions (Figure 2). Four facilities (score of 4) (CMH, LTH, NTH, and CCH) had standard treatment guidelines though not reviewed and updated with new evidence periodically. Further, advice or feedback from the AMS team was only fully accessible in three facilities (KGH, NTH, and MGH) and partially (score of 3) in three facilities (KTH, LTH, CCH). Only KGH and MGH conducted regular audits of specified antibiotic therapy or clinical conditions. Additionally, the AMS teams did not conduct ward rounds or other AMS interventions in selected departments across four facilities (KTH, LTH, CMH, and ADH). Besides, most facilities (88%, 7/8) namely LTH, KTH, KGH, CMH, CCH, MGH, and ADH did not have formularies that list antibiotics according to the AWaRe classification that requires approval from a designated person or team, thereby increasing the risks of irrational prescribing. Of concern, 62.5% (5/8) of the facilities (KTH, CMH, ADH, CCH, and LTH) did not have standardized prescription charts, medical records, patient folders, and transfer notes to support treatment and AMS activities. All the facilities except CCH performed well (score of 4) in ensuring access to laboratory and imaging services that supported AMS interventions. Besides, only three facilities (NTH, KGH, and MGH) had information technology services or other inventory control tools. Finally, 63%, 5/8 of facilities (KTH, NTH, ADH, CCH, and LTH) did not have a written policy that required prescribers to document the indications and antibiotics prescribed in a prescription chart or medical records (Figure 4).

### Education and training of healthcare workers on AMS in surveyed hospitals

The assessment was specifically centred on enhancing the inclusion of AMS programs in staff induction training and continuous professional development on AMS and IPC covering programs such as optimizing antibiotic prescribing, and dispensing.

Our findings revealed that 37.5% (3/8) of the facilities (KTH, CMH, and LTH) scored below average (54%) concerning the education and training of staff on AMS (Figure 2), indicating that most HCWs were not trained on AMS. Only two (ADH, and MGH) of the eight facilities partially included AMS programs such as optimizing antibiotic prescribing, dispensing, and administration in the staff induction training. Further, only two facilities (KGH, and MGH) fully offered continuous in-service training or continuous professional development on AMS and IPC to staff.

### Monitoring and surveillance of AMR in surveyed hospitals

This was evaluated hinging on the following; the regular prescription audits, point prevalence surveys assessing the appropriateness of antibiotic prescribing, regular monitoring of shortages or stock-outs of essential antimicrobials, and compliance to the predetermined AMS intervention.

Therefore, based on the above indicators, three (KTH, CMH, and ADH) of eight hospitals did not conduct regular prescription audits, or point prevalence surveys to assess the appropriateness of antibiotic prescribing, whereas four facilities (LTH, NTH, KGH, and CCH) did so but not regularly. Impressively, 87.5% (7/8) of the facilities (KTH, CMH, KGH, MGH, LTH, CCH, and ADH) regularly monitored the shortages or stock-outs of essential antimicrobials. Two facilities (KGH and MGH) also fully monitored compliance with at least one specific AMS intervention.

### Reporting feedback within the healthcare facility regarding AMR and AMS

**Figure 5:**
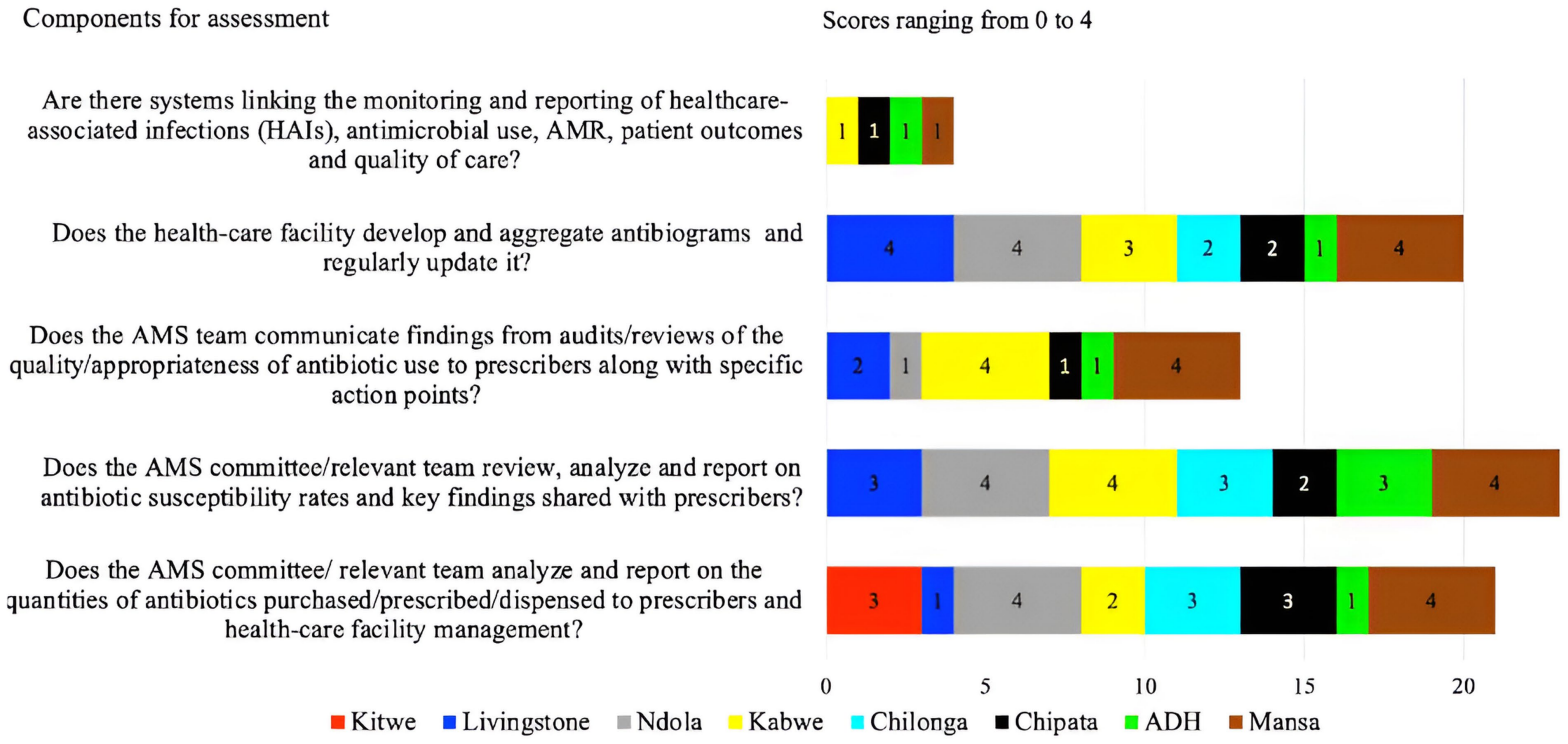
Scores of the eight facilities on their performance on reporting and feedback with the facility.

On reporting feedback, the assessment was based on the presence of reports on the quantities of antibiotics purchased, prescribed, and dispensed to prescribers. Additionally, the assessment considered whether systems were in place to link the monitoring and reporting of healthcare-associated infections (HAIs), antimicrobial use, AMR, patient outcomes, and quality of care. Further, the study assessed if the AMS teams communicated the findings from audits or reviews of the quality or appropriateness of antibiotic use to prescribers. The study found that more than half, 62.5% (5/8) of the facilities (ADH, CCH, KTH, CMH, and LTH) had challenges with reporting feedback within the healthcare facility and hence, performed poorly during the assessment. Most of the facilities scored below 50% with the highest and lowest being MGH (60%) and the lowest ADH (28%) (Figure 2). Two hospitals (NTH, and MGH) exhibited reporting on the quantities of antibiotics purchased, prescribed, and dispensed to prescribers. These two hospitals (NTH and KGH) also had evidence of antibiotic susceptibility rates and key findings being shared with prescribers (scored 4). Further, the assessment indicated that AMS teams from six facilities (LTH, KTH, CCH, CMH, ADH, and NTH) did not communicate their findings from audits or reviews of the quality/appropriateness of antibiotic use to prescribers along with specific action points. However, none (score of 1) of the hospitals assessed had systems linking the monitoring and reporting of healthcare-associated infections (HAIs), antimicrobial use, AMR, patient outcomes, and quality of care (see Figure 5).

### Challenges faced by public hospitals in implementing AMS programs in Zambia

Some of the challenges faced by healthcare facilities to implement AMS activities included a lack of leadership commitment, funding for AMS activities, dedicated human resources, inactive AMS multidisciplinary teams, lack of updated STGs, policies and tools, laboratory commodities, and ineffective communication between the AMS committees, prescribers and other health workers as shown in Table 3.

**Table 3:** Challenges faced by healthcare workers in implementing AMS activities.

AMS core element	Challenges faced by healthcare facilities in implementing AMS programs
Leadership commitment	Lack of leadership commitment to AMS Facility action plans have no AMS activities No funding for AMS activities No dedicated AMS leader with a job description for AMS
Accountability and responsibilities	No active AMS multidisciplinary team
AMS actions	Inadequate technical personnel needed for implementation of AMS Challenges/barriers in mechanisms of dissemination of AMS information No standard and updated treatment guidelines (STGs) in the facility Lack of AMS ward rounds and antibiotic review audit Lack of AWaRe tool for antibiotics Lack of facility AMS policy Lack of standardized prescription charts.
Education and training	The facility does not include AMS programs on optimizing antibiotic therapy, prescribing, dispensing, and administration of antibiotics. Inadequate training of staff on AMS
Monitoring and surveillance	Absence of antibiograms Lack of antibiotic sensitivity discs to effectively conduct surveillance No evidence-based practice from point prevalence surveys In hospitals that had antibiograms, they lacked regular updates due to poor surveillance
Reporting feedback	Inadequate communication on the resolutions of the DTC or AMS committees to the prescribers and other health workers

## Discussion

This study assessed the implementation of AMS activities in eight public hospitals in Zambia. The study found an average score of 56% (range: 41–78%) concerning the implementation of the WHO core elements for AMS across the selected healthcare facilities. This indicated that none of the assessed hospitals had fully functional AMS programs. Hence, they were not extensively benefiting from the positive impacts of well-functioning AMS programs such as reduced hospital costs, improved patient care, and treatment outcomes.

In comparison to the other assessed facilities, ADH, CMH, and KTH had the lowest overall scores, a difference that was attributed to the other facilities having established AMS programs with support from cooperating partners. This finding highlights the need and importance for each country to have a consistent government budget allocation for AMS implementation in their national yearly budget, unlike fully relying on cooperating partners, as shortage and lack of resources have been identified as a risk to the successful implementation of AMR and AMS programs (85).

The presence of the DTC or AMS teams had an overall score of 91%, however, most facilities did not have fully functional committees. Our score on the presence of the DTC and or AMS teams was higher than the set-up of AMS teams in healthcare facilities in South Africa which scored 75% (86). The higher score in Zambia was a result of the AMS teams being incorporated in the DTCs which have been part of the health care system for decades and act as formal channels of communication between medical staff and pharmacy department as well as policy-recommending bodies to medical personnel and hospital administration on the therapeutic use of medicines (87). Nevertheless, the low score in their functionality illuminates the need for improved AMS activities across the study sites. Our findings were similar to those reported in Kenya and Asia (88, 89). A non-functional DTC implies that the healthcare facilities do not have planned activities and allocated funds to implement AMS activities, hence, affecting the fight against AMR (90).

Our study revealed that only three of the eight hospitals had fully implemented the AWaRe classification of antibiotics. Similar findings have been reported across some WHO-African countries where gaps in implementing the national core elements for sustainable antimicrobial use and the WHO AWaRe classification of antibiotics were observed (91). The AWaRe classification of antibiotics is important in promoting rational prescribing and the use of antibiotics in healthcare facilities (59, 60, 92). A lack of implementation of the AWaRe tool may lead to inappropriate prescribing of antibiotics (93, 94). Evidence has indicated that the lack of implementation of the AWaRe tool in hospitals could be due to inadequate awareness among healthcare workers (95). Hence, increasing awareness and education about the AWaRe framework of antibiotics can improve adherence levels and rational prescribing (95, 96). Additionally, the low implementation of AMS programs in hospitals can affect the rational use of antibiotics (91).

This study found that most hospitals did not implement AMS programs that met the gold standard for AMS programs in hospitals as most of the core elements were not fully addressed. These findings corroborate reports from India where most healthcare facilities did not fully implement AMS programs (33). Similar findings were reported in a study conducted across 10 countries where AMS programs in hospitals did not meet the gold standard for AMS programs (89). A study conducted in 47 WHO-African countries found that most countries had many gaps in implementing the core elements of AMS (91). These findings require urgent attention because AMS programs are part of the recommendations of the Global Action Plan (GAP) and National Action Plans (NAPs) on AMR that aim to curb AMR using a One Health approach (37, 82). Therefore, there is a need to establish and strengthen AMS programs in the hospitals that scored low. This will optimize antibiotic use and improve patient outcomes (22, 32, 36, 76, 97, 98).

Our study found that leadership commitment to AMS was moderate, in that there was no allocation of necessary human resources and finances to implement AMS activities. It is critical to note that leadership commitment and accountability are essential in establishing and implementing effective AMS programs in hospitals (99, 100). This is because hospital leaders are in charge of providing support to instigate and run successful AMS programs (101). Despite the leadership commitment being just above 50% in our study, it was higher than that reported in South Africa (86). A lack of leadership commitment in Kenya prevented the establishment of robust AMS programs in hospitals (102). Leadership commitment has been reported in other studies to be essential for a successful AMS program as its absence generally affects responsibility, accountability, and commitment to AMS activities (99, 100, 103). Hence, improved leadership would facilitate the implementation of quality and sustainable AMS programs (99).

Interestingly, our study found that AMS core elements with high scores included monitoring and surveillance, accountability, and responsibility in addition to the presence of a DTC or an AMS team at the facility. These findings are encouraging as they explicate the potential for successful AMS implementation in these public health facilities. Our findings corroborate those that were reported in Nigeria where healthcare facilities had DTCs in place which were considered as facilitators of AMS (39). However, a study in Pakistan reported that despite the presence of a DTC, most antimicrobials were inappropriately prescribed thereby indicating the need for the establishment of AMS programs requiring accountability, policies, and responsibilities to address AMR (30). Therefore, the present DTCs can be strengthened to develop and coordinate rational medicines utilization, including AMS programs, and ensure that all activities run effectively (37, 73, 103).

Our study revealed that most facilities (62.5%) had challenges in reporting AMS feedback within healthcare facilities. This showed that the information from the DTC/AMS committees was not conveyed to the prescribers and other end users which could be probably a result of the non-functioning DTC committees in the facilities. A lack of AMS feedback in healthcare facilities could be due to a lack of enforcement and mechanisms for reporting and feedback (104). This in turn has negative implications on the implementation of AMS programs which may affect the prudent use of antibiotics and hence patient care. Local feedback on AMR or AMS activities is important to guide the prescribing of antibiotics (105) which in turn improves patient outcomes (106).

The current study of the sampled healthcare facilities revealed that all healthcare facilities had various challenges in implementing AMS programs. Our study found that all the facilities faced challenges regarding dedicated funding for AMS activities in their institutions. This could be attributed to the non-existence of AMS action plans and their subsequent inclusion in the overall hospital action plans. Funds are critical in ensuring that all costs for AMS activities are met. Lack of funding for AMS activities has been reported in other studies conducted in various countries (90, 91, 100, 107). The lack of locally generated antibiograms in the selected hospitals was another barrier to the effective implementation of successful AMS programs that depend on empirical microbiology data to inform rational prescribing of antibiotics in clinical use. Laboratory capacity challenges and lack of antibiograms have been widely reported as barriers to implementing AMS programs in hospitals in India, Kenya, Zambia, and Burkina Faso (90, 108–112). The absence of well-functioning laboratories that generate data for antibiograms negatively affects diagnostic stewardship and patient outcomes (108, 113, 114). Adequate laboratory capacity to detect resistant microbes leads to good clinical practice and promotes AMS.

In this study, the low scores in most of the AMS core elements reported point to the current weaknesses in the functionality and leadership roles of DTC and AMS committees. The functionality of the DTC and AMS committees is urgently required to instigate and sustain AMS programs at the hospital level (104). DTC/AMS committees must play key anchor roles to cascade national action plans at the hospital level, including forward-feed local hospital actions and data into the national AMS pipeline. We contend that to successfully implement AMS programs, making available dedicated domestic resources (fiscal, infrastructure, and human resources) can operationalize the concerted efforts needed to activate the multidisciplinary AMS teams to work together at the service delivery points. The institutional DTC and AMS committees and national AMS programs are supposed to drive the AMS actions in a coordinated manner. The lack of this coordinated approach may ultimately affect the incidence of AMR in the country. In addition, AMR and AMS should be mainstreamed in the health system development plan to drive capacity-building and behavioural change across the professional landscape, including addressing supply chain and regulatory challenges that are driving the irrational use of antimicrobials. Thus, the study recommends the establishment and/or strengthening of the DTCs/AMS committees to anchor AMS programs in hospitals across all levels of healthcare provision in the country.

We are aware of the study’s limitations. Since this study was conducted in selected secondary and tertiary hospitals in Zambia, the findings cannot be generalized to other hospitals. Additionally, the study used purposive sampling methods which is a non-probability sampling method thereby affecting the performance of statistical inferences, hence, calling for a more comprehensive study involving a representative number of hospitals across the country chosen randomly. However, our study demonstrates the capacity of hospitals to implement AMS activities in Zambia. Therefore, the identified gaps can be used to strengthen AMS activities in secondary and tertiary hospitals in Zambia to optimize the use of antibiotics in hospitals.

## Conclusion

This situational analysis study revealed critical gaps in AMS core elements across the selected hospitals in Zambia. The low DTC functionality, non-performance of AMS actions, lack of education and training on AMR and AMS, poor reporting of AMS feedback, and limited leadership commitment to AMS activities within healthcare facilities contributed to the non-performance of AMS programs. Additionally, inadequate finances for implementing sustainable AMS programs affected all healthcare facilities. There is an urgent need to mobilize domestic funding, design targeted interventions for AMS programs in healthcare facilities, and build capacity among healthcare workers and hospital leadership regarding all AMS core elements.

## Data Availability

The original contributions presented in the study are included in the article/Supplementary material, further inquiries can be directed to the corresponding author.
