# Supplementary material for: Antimicrobial stewardship situation analysis in selected hospitals in Zambia: findings and implications from a national survey
Source: Front Public Health. 2024 Sep 27;12:1367703. doi: 10.3389/fpubh.2024.1367703 (PMC11466898; doi:10.3389/fpubh.2024.1367703)
Supplement: Supplementary file 1 [file Table_1.docx]

**Supplementary Material:** The following supporting information has been attached; Table S1: Summarized information from the assessment highlighting strengths, weaknesses, opportunities, and challenges (SWOC) for each facility.

1. Arthur Davison Children’s Hospital SWOC

| STRENGTHS | WEAKNESSES |
| --- | --- |
| Leadership commitment   - The presence of DTC and AMS team (Though no IPC committee)   AMS Actions   - Access to laboratory imaging services   Education and Training   - The facilities include AMS programs in staff induction training - The facility offers IPC training to staff   Monitoring and Surveillance   - Monitoring of antibiotics susceptibility and AMS.   Reporting and Feedback   - Analyse and report antibiotic susceptibility rates and key findings to prescribers | Leadership commitment   - AMS activities not included in the facility action plans - No allocation of funding and staff for AMS - No mechanisms for monitoring AMR/AMS   Accountability and responsibility   - Lack of a multidisciplinary team for AMS - No terms of reference for the AMS team - No regular AMS committee meetings - No dedicated AMS leader with a job description for AMS - No clear terms of reference for the AMS team   AMS Actions   - No STGs in the facility - Lack of AMS ward rounds and antibiotic review audit - Lack of AwaRe tool for antibiotics - Lack of facility AMS policy - No IPC committee   Education and Training   - No training of staff on AMS   Monitoring and Surveillance   - No prescription audits, or PPS undertaken by the AMS committee   Reporting and Feedback   - No analysis and reporting of the quantities of antibiotics purchased, prescribed and dispensed - No antibiograms - No systems linking monitoring and reporting of healthcare-associated infections, antimicrobial use, AMR, patient outcome and quality of care |
| OPPORTUNITIES | CHALLENGES |
| Leadership   - AMS identified as a priority by the hospital leadership - Support from AMRCC to establish AMS   AMS Actions   - Availability of support from funders - Adoption of facility treatment guidelines from the University Teaching Hospital   Education and Training   - - It is a training institution   - Availability of AMR online courses | Leadership commitment   - Lack of leadership commitment - Facility action plans have no AMS activities - No funding for AMS activities   Accountability and responsibility   - No active AMS multidisciplinary team - No dedicated AMS leader with a job description for AMS   Education and Training   - Inadequate staff trained in AMS |

1. Chipata Central Hospital

| STRENGTHS | WEAKNESSES |
| --- | --- |
| Leadership commitment   - The presence of a DTC and AMS team (though IPC was not fully functional) - Having AMS activities on the annual action plans   Accountability and responsibility   - Presence of a multidisciplinary team for AMS although partially implemented - Holding regular AMS meetings   AMS Actions   - The AMS team has developed standardized prescription charts/ patient medical records   Education and Training   - The facility has made efforts in training healthcare workers on IPC and AMS   Monitoring and Surveillance   - The facility regularly monitors essential antimicrobial shortages - Presence of a mechanism to monitor substandard and falsified antimicrobials   Reporting and Feedback   - The AMS team has been making efforts to report the quantities of antibiotics purchased/prescribed/dispensed to prescribers and facility management | Leadership commitment   - No dedicated financial support for AMS - No budget for AMS activities   Accountability and responsibility   - No terms of reference for the AMS team - AMS leader does not have dedicated staff time for AMS activities in their terms of reference/job description - No hospital epidemiologist   AMS Actions   - No local facility treatment guidelines - Gaps in using the AWaRe Classification of antibiotics   Education and Training   - The facility has not been including AMS programs on optimizing antibiotic therapy, prescribing, dispensing, and administration   Monitoring and Surveillance   - AMS team does not monitor antibiotic susceptibility and resistance rates for a range of key indicator organisms   Reporting and Feedback   - AMS team does not communicate findings from audits, reviews of quantity/appropriateness of antibiotic use to prescribers along with specific action plans   - The facility does not have antibiograms due to a lack of antibiotic discs - The facility has no systems linking monitoring and reporting of healthcare-associated infections, antimicrobial use, AMR, patient outcome and quality of care |
| OPPORTUNITIES | CHALLENGES |
| Leadership   - AMS leadership can lobby for funds from many funders - The AMS team can lobby for support from the AMRCC - The government is willing to support all health-related programs, hence, the AMS team can request any resources needed to promote the rational use of antimicrobials   Accountability and responsibility   - - Available education for AMS staff globally   - Terms of reference can be adopted from the AMRCC   AMS Actions   - - Availability of support from funders   - Adoption of facility treatment guidelines from tertiary hospitals   Education and Training   - - Training on AMS is done sometimes during workshops outside the facility   Monitoring and Surveillance   - - AMS team can adopt some guidelines on monitoring and surveillance from other facilities like the UTH   Reporting and Feedback   - - The AMS team can engage external data analysts to help with the analysis and reporting of AMR data | Leadership commitment   - No funding for AMS activities - Budget challenges for AMS activities - Challenges in implementing the AMS activities   Accountability and responsibility   - Lack of training epidemiologists and other AMS team members   Education and Training   - Non-trained AMS staff   Monitoring and Surveillance   - - Lack of antibiotic discs   - No antibiogram to effectively conduct surveillance   Reporting and Feedback   - Challenges/barriers in mechanisms of dissemination of AMS information |

1. Kabwe General Hospital

| STRENGTHS | WEAKNESSES |
| --- | --- |
| Leadership commitment   - AMS is a priority at the facility - AMS activities - Adequate number of pharmacists in the AMS team - Presence of a mechanism that regularly monitors and measures the implementation of the AMS activities - The presence of a DTC or AMS team   Accountability and responsibility   - Presence of a multidisciplinary team on AMS with clear terms of reference - Regular AMS team meetings - Presence of AMS leader with clear terms of reference - Presence of the AMS team with clear terms of reference - Involvement of other healthcare workers in AMS activities other than the AMS team - Presence of an AMS team representative on the IPC coordinating team and vice-versa - AMS team generates reports of AMS implementation - AMS team disseminates AMS activity reports to the management and other healthcare workers at the facility   AMS Actions   - Presence of regular audits or reviews on specified antibiotic therapy or clinical conditions at the facility - Feedback from the AMS team easily accessible to prescribers - There is access to laboratory and imaging services at the facility - IT services are available at the facility that can be used to support data gathering that supports AMS activities - Presence of standardized patient prescription charts, folders, medical files, and transfer notes that support AMS activities - Presence of a policy that requires prescribers to indicate the indication of antibiotic therapy   Education and Training   - The facility offers CPD on AMS and IPC to the staff - The facility ensures the training of the AMS team on AMS and IPC   Monitoring and Surveillance   - The facility monitors the quantities and types of antibiotics purchased/ prescribed/ dispensed - The facility monitors stockouts/shortages of essential antibiotics - The facility monitors compliance with AMS interventions - The facility monitors antibiotic susceptibility and resistance rates for key indicator microbes   Reporting and Feedback   - The AMS team reviews, analyses and reports antibiotic susceptibility rates and key findings shared with prescribers - The AMS communicates findings from reviews/audits of the quality/appropriateness of antibiotic use to prescribers along with specific action points   Presence of systems for monitoring and reporting Healthcare-associated infections, antimicrobial use, AMR, patient outcomes and quality of care | Leadership commitment   - No financial support to AMS action plan - No budget to support the implementation of the AMS action pan   Accountability and responsibility   - No clinical pharmacist - No epidemiologist - No clinical microbiologist - No infection control practitioner   AMS Actions   - No facility STGs - Lack of AMS ward rounds and other AMS interventions in selected departments - No facility-approved formulary/list of antibiotics based on the national formulary - No AWaRe classification of antibiotics   Education and Training   - The facility does not include AMS programmes on optimizing antibiotic therapy, prescribing, dispensing, and administration of antibiotics   Monitoring and Surveillance   - Gaps in prescription audits, or PPS undertaken by the AMS committee to monitor the appropriateness of antibiotic use - Gaps in the mechanisms to monitor and report falsified and substandard antibiotics   Reporting and Feedback   - Gaps observed in the analysis and reporting of the quantities of antibiotics purchased/ prescribed/ dispensed - Gaps in regular updating of antibiograms - Financial challenges to support AMS activities - AMS personnel shortages |
| OPPORTUNITIES | CHALLENGES |
| Leadership   - AMS action plans can be adopted from higher hospitals like the UTH - Funding for AMS is available and can be accessed through grant applications   Accountability and responsibility   - - Education and CPD on AMS are available in many institutions and online   - The facility can facilitate sponsoring AMS team members to further their education concerning AMS   AMS Actions   - - AWaRe classification of antibiotics is readily available on the WHO website   - Facility treatment guidelines from tertiary hospitals   - A list of antibiotics to use at the facility can be adopted from the national formulary   Education and Training   - - Educational programs on AMS can be accessed from training institutions   - Online education activities on AMS are available   Monitoring and Surveillance   - The WHO guides how to conduct PPS and prescription audits   Reporting and Feedback   - The AMS team can adopt guidelines on how to analyze and report the quantities of antibiotics purchased/prescribed/dispensed | Leadership commitment   - No funding for AMS activities - No funds were allocated to support the AMS action plan - Lack of action plan that prioritises AMS activities   Accountability and responsibility   - No regular meetings on AMS activities - No trained personnel in AMS   Education and Training   - Lack of training on AMS among staff   Monitoring and Surveillance   - No evidence-based practice from PPS   Reporting and Feedback   - Poor surveillance due to a lack of regularly updated antibiograms - Lack of reporting on antibiotics purchased/prescribed/dispensed - Lack of funding support for surveillance activities and reporting |

1. Chilonga Mission General Hospital

| STRENGTHS | WEAKNESSES |
| --- | --- |
| Leadership commitment   - The presence of DTC and IPC team - Some pharmacists trained in AMS.   Accountability and responsibility   - Members of multi-displinary AMS committee identified.   AMS Actions   - Access to laboratory imaging services - Presence of evidence-based facility STG. - Presence of formulary list of approved antibiotics. - Facility AMS policy available.   Monitoring and Surveillance   - Monitoring of quality of antibiotics and stock out. - Monitoring of selected pathogen susceptibility profile. And resistance rates.   Reporting and Feedback  Analyze and report antibiotic susceptibility rates and key findings to prescribers | Leadership commitment   - AMS activities not included in the facility action plans - No allocation of funding and staff for AMS - No mechanisms for monitoring AMR/AMS   Accountability and responsibility   - Lack of a multidisciplinary team for AMS - No terms of reference for the AMS team - No regular AMS committee meetings - No dedicated AMS leader with a job description for AMS - No clear terms of reference for the AMS team - Lack of collaboration with other programs ie IPC, TB, HIV.   AMS Actions   - Lack of AMS ward rounds and antibiotic review audit - Lack of AwaRe tool for antibiotics - Lack of AWaRe categorization and antibiotic restriction.   Education and Training   - No training of staff on AMS and IPC at indcution - Lack of CPD on AMS and IPC.   Monitoring and Surveillance   - No prescription audits, or PPS undertaken by the AMS committee - Lack of monitoring of AMS interventions.   Reporting and Feedback   - No analysis and reporting of the quantities of antibiotics purchased, prescribed and dispensed - No systems linking monitoring and reporting of healthcare-associated infections, antimicrobial use, AMR, patient outcome and quality of care |
| OPPORTUNITIES | CHALLENGES |
| - AMS identified as a priority by the hospital leadership and members already identified. - Support from AMRCC to establish AMS   - Sentinel site for laboratory surveillance.   - Strengthen use of facility antimicrobial susceptibility data to update STG | - Facility action plans have no AMS activities - No funding for AMS activities - No active AMS multidisciplinary team - No dedicated AMS leader with a job description for AMS - Inadequate staff trained in AMS |

1. Livingstone Teaching Hospital

| STRENGTHS | WEAKNESSES |
| --- | --- |
| Leadership commitment   - The presence of DTC or AMS team - Action plan approved by management. - Medicines use policy and procedure in place. - AMS identified as a priority and activities included in facility action plan. - Financial and human resource allocated to initiate AMS activities. - Regular monitoring of AMS activities.   AMS Actions   - Access to laboratory imaging services - Regular clinical audits on specific conditions and antimicrobial therapy.   Education and Training   - The facilities include AMS programs in staff induction training - The facility offers CPD on IPC/AMS training to staff.   Monitoring and Surveillance   - Monitoring of antibiotics susceptibility and AMS. - Regular PPS and prescription audit undertaken by AMS committee. - Monitoring of quality and supply chain of essential antimicrobials.   Reporting and Feedback   - Analyze and report antibiotic susceptibility rates and key findings to prescribers - Report analysis of the quantities of antibiotics purchased, prescribed and dispensed | Leadership commitment   - Policy on antimicrobial use not available. - Lack of budget line for AMS activities on action plan.   Accountability and responsibility   - Lack of a multidisciplinary team for AMS - No terms of reference for the AMS team - No regular AMS committee meetings - No dedicated AMS leader with a job description for AMS - No clear terms of reference for the AMS team   AMS Actions   - No STGs in the facility - Lack of AMS ward rounds and antibiotic review audit - Lack of AwaRe tool for antibiotics - Lack of facility AMS policy - Lack of standardized prescription charts.   Monitoring and Surveillance   - Lack of monitoring of susceptibility resistance pattern for selected pathogens.   Reporting and Feedback   - Lack of consistent reporting of antibiograms - No systems linking monitoring and reporting of healthcare-associated infections, antimicrobial use, AMR, patient outcome and quality of care |
| OPPORTUNITIES | CHALLENGES |
| - AMS identified as a priority by the hospital leadership - Support from AMRCC to establish AMS | - Lack of leadership commitment - No sustainable funding for AMS activities - No active AMS multidisciplinary team - No dedicated AMS leader with a job description for AMS - Inadequate staff trained in AMS |

1. Kitwe Teaching Hospital

| STRENGTHS | WEAKNESSES |
| --- | --- |
| Leadership commitment   - The presence of DTC, AMS and IPC teams - AMS identified as a priority by management - Staff allocated for AMS   Accountability and responsibility   - DTC/AMS meets regular basis - Other health-care professionals are part of the AMS team   AMS Actions   - Access to laboratory imaging services - Reviews/audits of the antibiotic therapy - Facility has formulary/list of approved antibiotics for use based on the national formulary   Education and Training   - The facilities include AMS programs in staff induction training - The facility offers IPC training to staff   Monitoring and Surveillance   - Monitoring of antibiotics susceptibility and AMS   Reporting and Feedback   - Monitors the quantity and types of antibiotic use (prescribed/dispensed/purchase) | Leadership commitment   - DTC members have no official appointment letters - DTC/AMS has no action plan approved by management - AMS activities not included in the facility action plans - No allocation of funding for AMS - No mechanisms for monitoring AMR/AMS   Accountability and responsibility   - Lack of a multidisciplinary team for AMS with clear TORs - No dedicated AMS leader with a job description for AMS - No clear terms of reference for the AMS team   AMS Actions   - No STGs in the facility - Lack of AwaRe tool for antibiotics - No ward rounds on AMS - Lack of facility AMS policy - No standardized prescription charts   Education and Training   - The facilities include AMS programs in staff induction training - No training of staff on AMS     Monitoring and Surveillance   - No prescription audits, or PPS undertaken by the AMS committee   Reporting and Feedback   - No analysis and reporting of antibiotic susceptibility rates and key findings to prescribers - No antibiograms - No systems linking monitoring and reporting of healthcare-associated infections, antimicrobial use, AMR, patient outcome and quality of care |
| OPPORTUNITIES | CHALLENGES |
| Leadership   - AMS identified as a priority by the hospital leadership - Support from AMRCC to establish AMS   AMS Actions   - - Availability of support from funders   - Adoption of facility treatment guidelines from the University Teaching Hospital   Education and Training   - - It is a training institution   - Availability of AMR online courses | - Lack of leadership commitment - Facility action plans have no AMS activities - No funding for AMS activities - No dedicated AMS leader with a job description for AMS - Inadequate staff trained in AMS |

1. Ndola Teaching Hospital

| STRENGTHS | WEAKNESSES |
| --- | --- |
| Leadership commitment   - The presence of DTC, AMS and IPC teams (though IPC not fully functional) - AMS identified as a priority by management - Staff allocated for AMS   Accountability and responsibility   - DTC/AMS meets regular basis - Other health-care professionals are part of the AMS team - AMS team produce regular activity reports and disseminate to health workers   AMS Actions   - Access to laboratory imaging services - Reviews/audits of the antibiotic therapy - Facility has formulary/list of approved antibiotics for use based on the national formulary - Conduct regular ward rounds on AMS - Presence of standardized prescription charts   Education and Training   - The facility offers IPC training to staff - Trained staff on AMS   Monitoring and Surveillance   - Monitor the quantity and types of antibiotic use(purchased/prescribed/dispensed) - Monitor of antibiotics susceptibility and AMS. - Regular monitor the shortages/stock-outs of essential antimicrobials   Reporting and Feedback   - Analyze and report on the quantities of antibiotic use (prescribed/dispensed/purchase) - Analyze and report on antibiotic susceptibility rates and key findings to prescribers   Antibiograms available | Leadership commitment   - DTC members have no official appointment letters - DTC has no TORs - DTC/AMS has no action plan approved by management - AMS activities not included in the facility action plans - No allocation of funding for AMS - No mechanisms for monitoring AMR/AMS   Accountability and responsibility   - Lack of a multidisciplinary team for AMS with clear TORs - No dedicated AMS leader with a job description for AMS - No clear terms of reference for the AMS team   AMS Actions   - No STGs in the facility - Lack of AwaRe tool for antibiotics - Lack of facility AMS policy   Education and Training   - The facilities include AMS programs in staff induction training   Monitoring and Surveillance   - No prescription audits, or PPS undertaken by the AMS committee   Reporting and Feedback   - No systems linking monitoring and reporting of healthcare-associated infections, antimicrobial use, AMR, patient outcome and quality of care |
| OPPORTUNITIES | CHALLENGES |
| Leadership   - AMS identified as a priority by the hospital leadership - Support from AMRCC to establish AMS   AMS Actions   - Availability of support from funders   Education and Training   - It is a training institution - Availability of AMR online courses | - Facility action plans have no AMS activities - No funding for AMS activities - No dedicated AMS leader with a job description for AMS - Inadequate staff trained in AMS |

1. Mansa General Hospital

| STRENGTHS | WEAKNESSES |
| --- | --- |
| Leadership commitment   - The presence of DTC, AMS and IPC committees. - DTC action plan approved by management. - Medicine use policy and procedure available. - AMS activities included in the facility action plans - Allocation of funding and staff for AMS - Mechanisms for monitoring AMR/AMS in place   Accountability and responsibility   - terms of reference for the AMS team in place - Regular AMS committee meetings held. - Dedicated AMS leader with a job description for AMS.   AMS Actions   - Access to laboratory imaging services - STGs available in the facility - AMS ward rounds and antibiotic review audit routinely conducted. - AwaRe tool for antibiotics implemented - facility AMS policy developed.   Education and Training   - The facilities include AMS programs in staff induction training - The facility offers IPC and AMS CPD training to staff   Monitoring and Surveillance   - Monitoring of antibiotics susceptibility and AMS. - Prescription audits, or PPS undertaken by the AMS committee - Monitor quality and supply chain of essential antimicrobials. - Monitors antibiotic susceptibility and resistance for selected organisms.   Reporting and Feedback   - Analyze and report antibiotic susceptibility rates and key findings to prescribers. - Analysis and reporting of the quantities of antibiotics purchased, prescribed and dispensed - · Antibiograms developed and shared by microbiology laboratory. | Accountability and responsibility   - Lack of a complete multidisciplinary team for AMS   Reporting and Feedback   - No systems linking monitoring and reporting of healthcare-associated infections, antimicrobial use, AMR, patient outcome and quality of care |
| OPPORTUNITIES | CHALLENGES |
| - AMS identified as a priority by the hospital leadership - Availability of support from management | - No funding for AMS activities - Inadequate staff trained in AMS - Inadequate technical personnel need for implementation of AMS. |
